# Forecasting Artificial Intelligence Trends in Health Care: Systematic International Patent Analysis

**DOI:** 10.2196/47283

**Published:** 2023-05-26

**Authors:** Stan Benjamens, Pranavsingh Dhunnoo, Márton Görög, Bertalan Mesko

**Affiliations:** 1 Department of Surgery Ikazia Hospital Rotterdam Netherlands; 2 The Medical Futurist Institute Budapest Hungary; 3 Department of Computing Donegal Campus Atlantic Technological University Letterkenny Ireland

**Keywords:** artificial intelligence, patent, healthcare, health care, medical, forecasting, future, AI, machine learning, medical device, open-access, AI technology

## Abstract

**Background:**

Artificial intelligence (AI)– and machine learning (ML)–based medical devices and algorithms are rapidly changing the medical field. To provide an insight into the trends in AI and ML in health care, we conducted an international patent analysis.

**Objective:**

It is pivotal to obtain a clear overview on upcoming AI and MLtrends in health care to provide regulators with a better position to foresee what technologies they will have to create regulations for, which are not yet available on the market. Therefore, in this study, we provide insights and forecasts into the trends in AI and ML in health care by conducting an international patent analysis.

**Methods:**

A systematic patent analysis, focusing on AI- and ML-based patents in health care, was performed using the Espacenet database (from January 2012 until July 2022). This database includes patents from the China National Intellectual Property Administration, European Patent Office, Japan Patent Office, Korean Intellectual Property Office, and the United States Patent and Trademark Office.

**Results:**

We identified 10,967 patents: 7332 (66.9%) from the China National Intellectual Property Administration, 191 (1.7%) from the European Patent Office, 163 (1.5%) from the Japan Patent Office, 513 (4.7%) from the Korean Intellectual Property Office, and 2768 (25.2%) from the United States Patent and Trademark Office. The number of published patents showed a yearly doubling from 2015 until 2021. Five international companies that had the greatest impact on this increase were Ping An Medical and Healthcare Management Co Ltd with 568 (5.2%) patents, Siemens Healthineers with 273 (2.5%) patents, IBM Corp with 226 (2.1%) patents, Philips Healthcare with 150 (1.4%) patents, and Shanghai United Imaging Healthcare Co Ltd with 144 (1.3%) patents.

**Conclusions:**

This international patent analysis showed a linear increase in patents published by the 5 largest patent offices. An open access database with interactive search options was launched for AI- and ML-based patents in health care.

## Introduction

Artificial intelligence (AI), in the form of machine learning (ML)–based medical devices and algorithms, has been rapidly changing a range of aspects of the medical profession from clinical decision-making to diagnostic imaging interpretation [1,2]. Both the commercial development and academic research focusing on AI and ML in health care showed an exponential growth; however, regulation for clinical use and commercial rollout follow a slower path.

The US Food and Drug Administration (FDA) has been leading the way for regulators worldwide, being the first regulatory body to adopt an AI policy and provide guidelines for approving AI-based medical technologies in practice [3].

Certain medical specialties stand out in terms of the impact of AI on the practice of those professions. Based on a previous study by our research group, examples include cardiology, radiology, and oncology—medical specialties that entail many data-based tasks and components. A better understanding of which medical specialties will be impacted by AI in the near future might shed light on what guidelines, policies, or frameworks to dedicate enough efforts to next.

Also, while the number of peer-reviewed papers on AI’s role in health care and medicine, relevant patents, and commercially available AI and ML devices keeps on growing at an unprecedented rate, it will become increasingly difficult for regulators and policy makers to keep up with the pace of innovation [4].

There are numerous health care– and AI-related patents worldwide. Inventors and researchers can submit their patents to national and international offices, of which the largest ones include the China National Intellectual Property Administration (CNIPA), the European Patent Office (EPO), the Japan Patent Office (JPO), the Korean Intellectual Property Office (KIPO), or the United States Patent and Trademark Office. These 5 largest patent offices collaborate in the Five IP Offices collaboration, making all their patents available in the Global Dossier initiative [5].

Not every patent will lead to a product or a service on the market, and even for those that succeed, it usually takes years to reach the market and end up being a commercially available product or a product used in the medical practice.

For example, a patent was submitted for “wireless transmission of ECGs in handheld devices” in 1998 in the United States [6]. The applicants of the patent developed the idea of a smartphone case that served as a single-lead electrocardiogram to be approved by the FDA in 2012, a total of 14 years later. The evolution of its design resulted in a credit card–sized device and an even smaller version in 2021. In the meantime, the company AliveCor received clearance by the FDA to use an algorithm for the analysis of the readings to determine issues related to cardiac rhythm without human intervention [7]. It took around 2 decades for a digital health technology to transition from a patent phase to becoming commercially available, and years to build AI analysis into the device.

It is pivotal to obtain a clear overview on upcoming AI and ML trends in health care to provide regulators with a better position to foresee what technologies they will have to create regulations for that are not available in the market yet.

Therefore, in this study, we provide insights and forecasts into the trends in AI and ML in health care by conducting an international patent analysis.

## Methods

### Selection of Patents

We selected the Espacenet search engine of the EPO to access the data from the 5 international patent offices collaborating in the Global Dossier initiative [8-10]. The Global Dossier initiative enables web-based public access for the patent data of the CNIPA [11], EPO, JPO [12], KIPO [13], the United States Patent and Trademark Office [14], and provides computer translations to English for the CNIPA, JPO, and KIPO.

We performed a systematic search for the period between January 1, 2012, and July 20, 2022. A query was made using the following keywords: *deep learning*, *machine learning*, *deep neural networks*, or *artificial intelligence*, in combination with *medical*, *medicine*, *healthcare*, or *health*. In addition, the “computing arrangements based on specific computational models” (G06N) of the Cooperative Patent Classification (CPC) was used [15]. Patents are classified with at least 1 CPC code and the G06N is assigned when the invention relates to AI or ML techniques.

The following variables were extracted from the Espacenet database: patent title and abstract, inventors, applicants, publication number, CPC code, and publication date. As inventors are allowed to submit their patent at multiple patent offices, duplicate patents were removed based on matching titles, inventor names, and applicant names. The patent publication number was used to identify the patent office that registered the patent.

### Downloading Patent Abstracts

In total, 12,384 matches were found using the Espacenet search, based on which we performed the analysis. Search queries might contain overlapping results; therefore, we excluded duplications, finally retaining 10,967 distinct matches.

Public information was downloaded for all of the patents using a Chrome-based crawler from Espacenet, followed by the extraction of titles and abstracts from the HTML source. The resulting text data were saved to files for further processing. Crawling was performed in August and September 2022.

### Preprocessing of Textual Data

The first publication date and number were used wherever multiple were available.

We retained patents that were dated after January 1, 2016, excluding 197 (1.79%) patents of the available data set. The last fully covered month was June 2022, the few patents (n=27, 0.25%) in July 2022 were excluded.

Some of the most frequent words of the English language were excluded from the analysis, as they would rank high in appearance statistics without highlighting the trends we are looking for. The excluded words were the following: “for,” “from,” “and,” “with,” “on,” “of,” “a,” “the,” “to,” “is,” “an,” “by,” “are,” “in,” “can,” “or,” “that,” and “be.” Additionally, commas and parentheses were removed, and the text was converted to lowercase.

### Statistics Generated From Downloaded Data

Multiple statistics were generated from the patents; these were evaluated separately for titles and abstracts as well, except for top lists.

#### Occurrence Counts: Single Words

Titles of all used patents were merged into 1 string, and the number of times each word appears was counted. The occurrence count list was constructed the same way for abstracts as well. Each word appearance was counted, not limited to 1 per patent.

#### Abstract Query

Some further cases were not covered by the abovementioned lists: expressions consisting of 2 or more words (eg, “brain ct image”), or words from the abstracts that are not listed above due to a very low occurrence count. A researcher could look up arbitrary texts using the query form.

After performing the preprocessing steps, the query string was looked up in each patent’s title. The number of patents with matches was counted—that is, each patent is counted once at maximum—as opposed to the “occurrence counts” described above.

Additionally, appearance counts were displayed on a time scale as well to visualize trends in 3-month units.

Furthermore, to eliminate the effect of increasing patent count, the relative frequency of the search term was also displayed—this is useful to determine the trends of methods because raw occurrence counts could increase even with a declining technology when total patent counts increase over time.

#### Top Lists

Inventor, applicant, and CPC top lists are simple lists with occurrence counts, based on patent properties without any pre- or postprocessing steps.

A list of the top 20 medical specialties and related terms was curated (Multimedia Appendix 1): anesthesiology, cardiology, dentistry, dermatology, emergency medicine, gastroenterology, gerontology, family medicine or primary care, internal medicine (ie, infectiology, endocrinology, and nephrology), neurology, obstetrics and gynecology, oncology, ophthalmology, pathology, pediatrics, psychiatry, pulmonology, radiology or nuclear medicine, surgery, and urology [16].

### Open Access, Interactive Database

We made our database open access, which is available on The Medical Futurist website [17]. The page allows visitors to analyze the patent database to validate our findings and discover other trends. The code is available upon request.

Users can select from among the available functions in the left sidebar, while the content for the chosen page appears on the right side.

In this web-based open access database, term frequency–inverse document frequency is applied for the purpose of frequency scoring. Single-word occurrences within titles and abstracts were introduced above, along with *Query* and *Toplists* pages. Besides these, the most frequent word pairs (eg, image segmentation) are also listed with the number of occurrences separately for titles and abstracts. Finally, under “Trending,” one can find those expressions whose occurrence rises steadily within the last 5 examined quarters, possibly highlighting methods that are currently becoming popular. The “Trending” page examines 3 separate properties: change in absolute and relative occurrence, along with the shape of the increase by quarters correlated to a linearly increasing line in the (the “Trend” column).

Interestingly, most of the single words with a high relative increase are linked to modern technologies within health care (“device,” “forecasting,” “inference,” and “classifying”). Similarly, some of the increasingly used word pairs are “computer aided” and “learning algorithms.”

## Results

By using the patent database filter option “Applicant toplist,” a list in descending order of the number of patent applications per applicant was generated. The number of patents applied by an entity ranged from 1 to 305, with applicant Ping An Technology (Shenzhen, China) filing for the highest number of patents (n=305) and several dozens of applicants filing for the lowest number of patents (n=1). We identified 5848 patents with a company as the primary applicant and 3038 patents with a university as the primary applicant. To derive insights relevant for the purposes of this study, the 20 applicants from this list, which applied for the most patents, were considered and the findings are summarized in Table 1.

Each entry in the “Applicant toplist” filter also lists the corresponding country, in abbreviated format, where the relevant patent office is located. Out of the top 20 patent applicants, 14 are based in China, 3 are based in the United States, and 1 is based in Germany, Japan, and the Netherlands, each.

From these data and extending to applicants beyond the top 20 ones, a list of the top 10 countries where most patents were applied from was curated. As Table 1 indicates, most of the relevant patent applications were filed in China, followed by the United States. Among this list of top 10 countries, 4 are located in Asia, 4 are located in Europe, and 2 are located in North America. Textbox 1 shows the top 10 countries from where relevant patents were filed.

**Figure 1:**
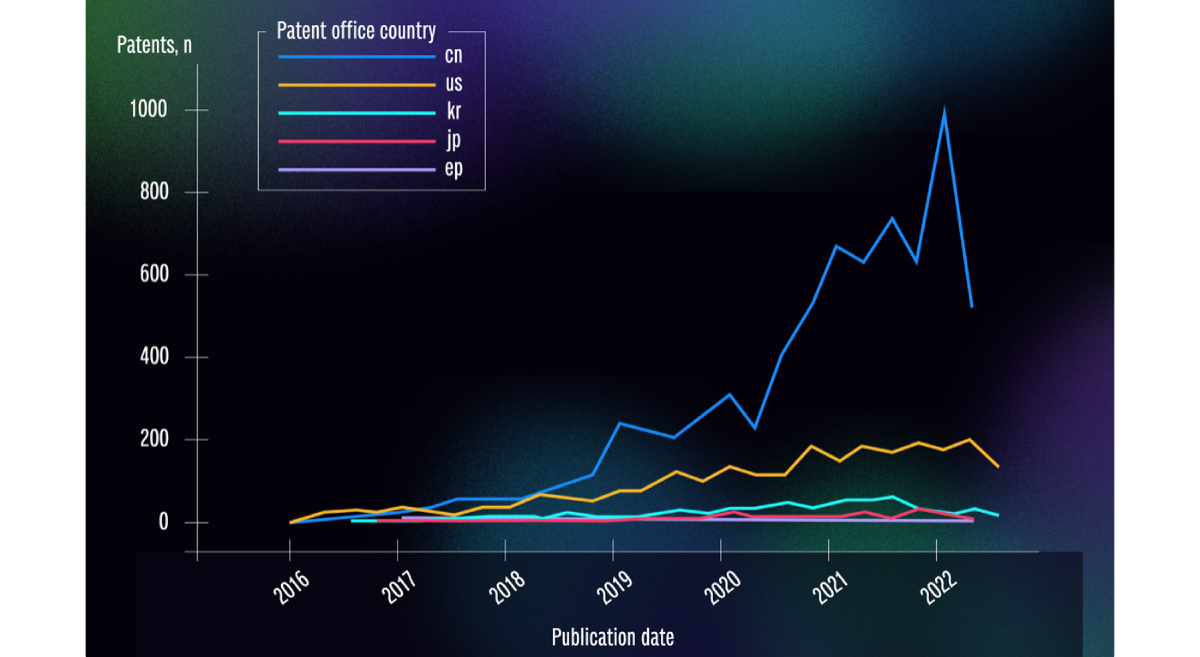
Patent trends in selected patent offices between 2016 and 2022. cn: China; ep: Spain; jp: Japan; kr: South Korea; us: United States.

By selecting the “Patent office stats” option from the database, the general trend in the number of health care patents between 2016 and 2022 in selected patent offices was observed. There were 156, 340, 747, 1552, 253, 4097, and 1278 AI- and ML-related health care patents in 2016, 2017, 2018, 2019, 2020, and July 2021, respectively; this indicates a general increase in the application of such patents during that time period in the patent offices in China, the United States, and South Korea, while the offices in Japan and Spain have experienced little to no change in the volume of patents. Figure 1, generated from the database, plots the number of patents in the selected patent offices over this time period.

**Table 1:** Top 20 patent applicants.

Number	Applicant name	Occurrences, n	Country
1	Ping An Technology (Shenzhen) Co Ltd	305	China
2	Siemens Healthcare GmbH	219	Germany
3	IBM Corp	217	United States
4	Koninklijke Philips N.V.	110	The Netherlands
5	Ping An Medical and Healthcare Management Co Ltd	105	China
6	Ping An International Smart City Technology Co Ltd	103	China
7	Tencent Technology Shenzhen Co Ltd	90	China
8	University of Electronic Science and Technology of China	82	China
9	Zhejiang University	79	China
10	Shandong University	59	China
11	Beijing University of Technology	57	China
12	Tsinghua University	57	China
13	Fudan University	50	China
14	Canon Medical Systems Corporation	47	Japan
15	Beijing Baidu Netcom Science Technology Co Ltd	46	China
16	Tianjin University	45	China
17	GE Precision Healthcare LLC	45	United States
18	Huazhong University of Science and Technology	45	China
19	Beihang University	44	China
20	General Electric	44	United States

**Top 10 countries from where patents were filed.**

The top 10 countries from where patents were filed were as follows:

1. China
2. United States
3. South Korea
4. Germany
5. Japan
6. The Netherlands
7. Canada
8. India
9. United Kingdom
10. France

The rate of increase in the number of patents varied in each office that experienced such an increase. A marked increase was noticeable in China from mid-2017, around 2018 in the United States, and only around 2020 in South Korea. The patent office in China experienced a steady increase in the number of applications with some notable dips in 2020, 2021, and 2022. Despite those downtimes, that patent office maintained its lead during the time period analyzed.

To analyze patent trends around medical specialties, we created a database of words and expressions that are relevant to each of the major 20 medical specialties (Multimedia Appendix 1).

When analyzing single words that appear in the title of patents, the top 5 medical specialties with the highest number of patents were radiology, oncology, cardiology, pulmonology, and surgery with 394, 271, 128, 103, and 76 patents, respectively.

The “Abstract - query” option of the database outputs the number of times the search term occurs in the abstracts. Using the preselected specific terms for medical specialties, the occurrence of specialty-related terms was identified. Based on this list, the terms relating to radiology or nuclear medicine occurred the most in abstracts (n=1160), followed by oncology (n=532), ophthalmology (n=454), surgery (n=309), pulmonology (n=261), cardiology (n=252), and obstetrics and gynecology (n=217; Table 2).

**Figure 2:**
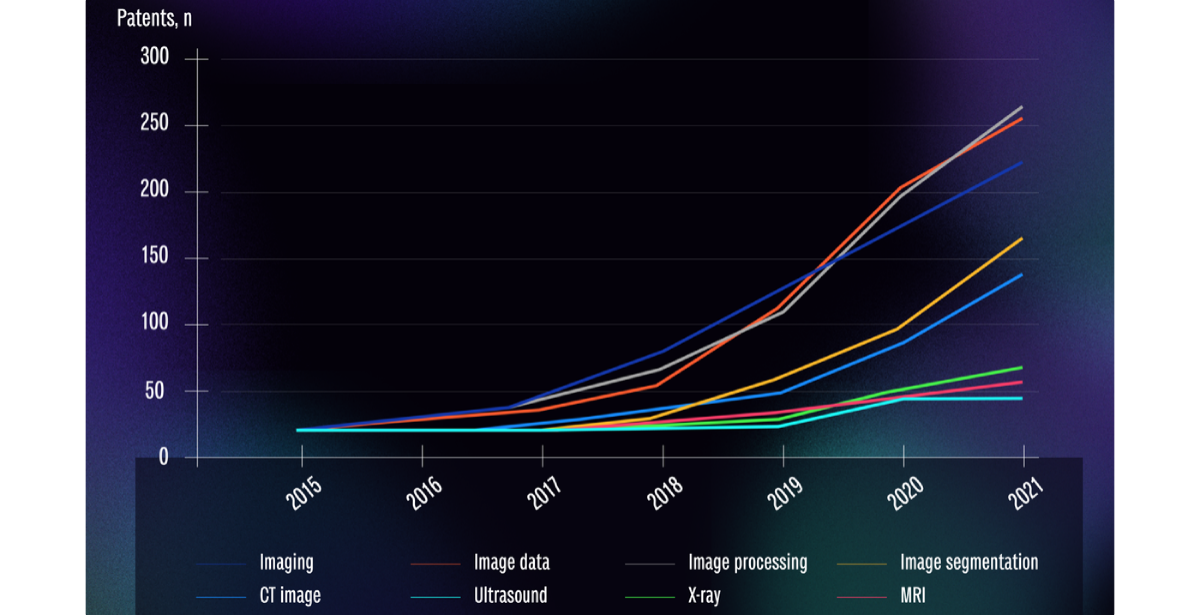
Trends in imaging-based patents. CT: computed tomography; MRI: magnetic resonance imaging.

When focusing on one of the medical specialties with a high number of patents (for instance, radiology), trends in imaging-based patents could be established. The 8 most frequently used imaging-related terms were “image processing” (n=682), “image data” (n=674), “imaging” (n=657), “image segmentation” (n=328), “CT image” (n=288), “X-ray” (n=120), “MRI” (n=114), and “ultrasound” (n=77). An increase in the occurrence of these imaging-related terms was identified between 2015 and 2021 (Figure 2). For the field of oncology, trends showed a similar increase. The 4 most used terms were “cancer” (n=161), “tumor” (n=151), “radiotherapy” (n=55), and “malignant” (n=47).

**Figure 3:**
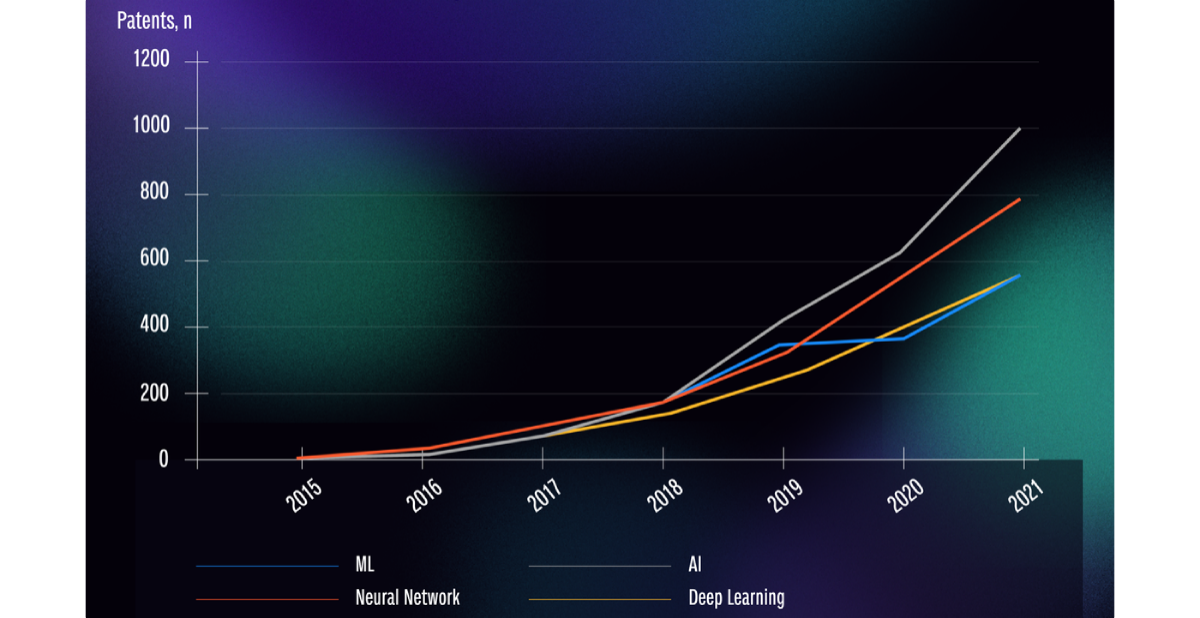
Trends in AI- and ML-based patents. AI: artificial intelligence; ML: machine learning.

When focusing on terms related to AI and ML, trends in AI- and ML-based patents could be established. The 4 most used AI- and ML-based terms were “artificial Intelligence” (n=2450), “neural network” (n=2043), “machine learning” (n=1717), and “deep learning” (n=1492). An increase in the occurrence of these AI- and ML-based terms was identified between 2015 and 2021 (Figure 3).

To demonstrate what kind of patents were included in the database, we chose to feature examples of recently registered patents of the top 4 applicants: Ping An Group listed a patent within the scope of the specialties of radiology and oncology, titled “Lymph node metastasis prediction method and device, equipment and storage medium” (CN113920137a) in January 2022. This patent focuses on the detection of lymph node metastasis in pancreatic ductal cancer on computed tomographic imaging of the abdomen. The results of the first clinical application were published in January 2023 [18].

Siemens Healthineers AG listed a patent within the scope of the specialties of radiology and pulmonology, titled “Assessment of abnormality patterns associated with covid-19 from x-ray images” (US2022022818a) in January 2022. A full package of AI solutions for COVID-19 imaging became commercially available the months thereafter [19].

IBM Corp listed a patent within the scope of the specialties of pathology and oncology, titled “Interpretation of whole-slide images in digital pathology” (US2022164946A1) in May 2022. The code, data, and models were published in January 2022 and a Python-based package (for modeling and learning) is freely available on GitHub [20,21].

Koninklijke Philips N.V. listed a patent within the scope of the specialty of cardiology, titled “Systems and methods for identifying low clinical value telemetry cases” (US2022020478A1) in January 2022. This patent is part of the Philips Cardiologs arrhythmias diagnostic software, which is commercially available and FDA-cleared under section 510(k) of the Food, Drug and Cosmetic Act [22].

**Table 2:** Occurrence of specialty-related terms.

Number	Specialty	Occurrences, n
1	Anesthesiology	72
2	Dentistry	41
3	Cardiology	252
4	Dermatology	112
5	Emergency medicine	157
6	Gastroenterology	84
7	Gerontology	37
8	Family medicine or primary care	28
9	Internal medicine	174
10	Neurology	77
11	Obstetrics and gynecology	217
12	Oncology (ie, radiation oncology)	532
13	Ophthalmology	454
14	Pathology	87
15	Pediatrics	18
16	Psychiatry	94
17	Pulmonology	261
18	Radiology	1160
19	Surgery	309
20	Urology	28

## Discussion

### Principal Findings

Based on the identified health care–related AI-based patents, China clearly stands out as a leader in the field of AI. Also, 2020 seems to be a turning point with marked growth in health care–related patents. Following the widespread success of ChatGPT (OpenAI) in 2022, there are no data to indicate that this growth would slow down [23].

**Figure 4:**
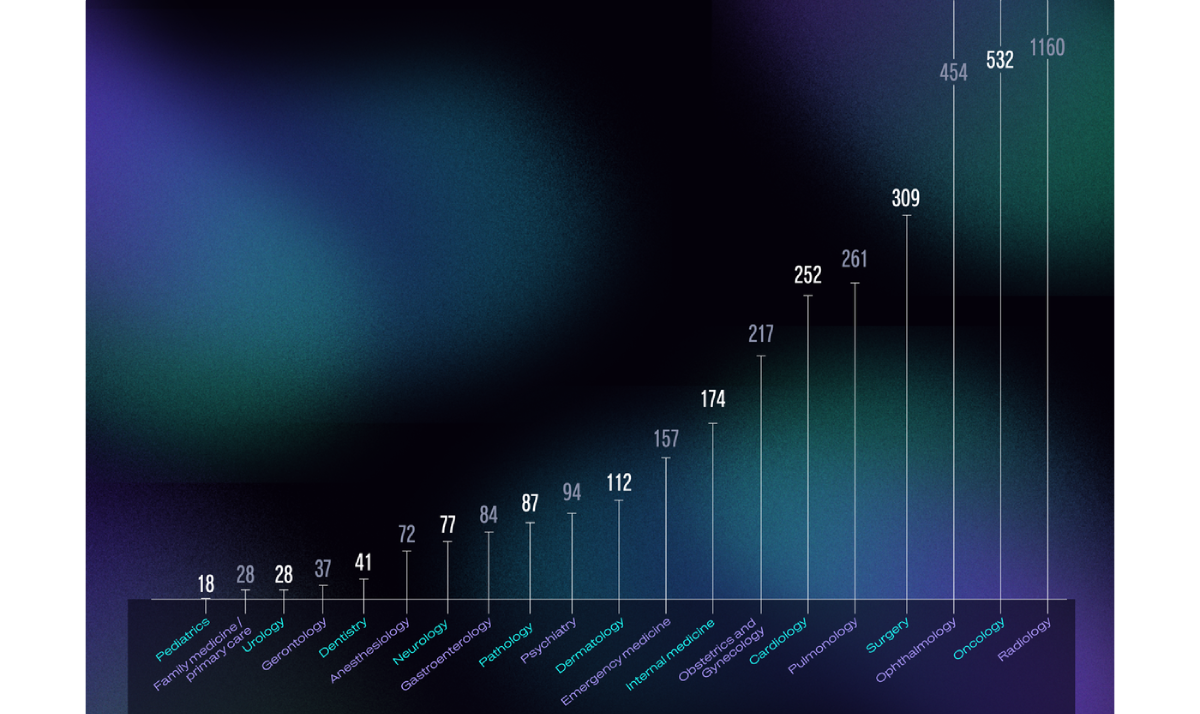
The number of occurrences of specialty-related terms in healthcare AI patents assigned to each of the 20 medical specialties. AI: artificial intelligence.

Certain medical specialties stand out in terms of the number of patents that have been submitted about AI technologies and inventions that might be relevant to them (Figure 4). Based on a previous study published by our group about FDA-approved AI- and ML-based medical technologies [1], radiology, cardiology, and oncology were already identified as specialties with many AI-based applications. The more repetitive or data-based tasks a specialty entails, the higher the potential for automation to be able to contribute to that field.

Moreover, patents that include the analysis of medical images or videos can be relevant to a range of specialties from radiology to pulmonology and surgery. Specialties that are closely linked to medical imaging can also be in the focus of AI patents in the coming years. Examples include dentistry, ophthalmology, and emergency medicine.

Besides these imaging-oriented specialties, as analyzing images is a widely popular use case of AI and ML, dermatology and pathology could also benefit from the AI revolution. In dermatology, the rise of skin-checking applications that can analyze photos of skin lesions on patients’ smartphones underscores this observation [24]. In pathology, automated assessment of digitized histopathology slides falls into the same category [25].

Medical specialties such as psychiatry or neurology that are more interaction-based (as opposed to being data-based) and entail more creative (vs repetitive) tasks might receive fewer AI patents; thus, those could be less prone to AI- or ML-based innovations [26].

The discrepancy between the top-ranking medical specialties in the title and abstract analyses could be attributed to the higher occurrence of related terms in the abstracts than in the titles, given the higher density of words in the former.

With this study, we attempt to prove the point that in the age of automation, preparing with regulations in time should be of high priority among policy makers. The #wearenotwaiting movement that comprises thousands of patients with diabetes, who created artificial pancreatic systems, further emphasizes this [27]. These patients have developed applications, platforms, and other solutions to help each other manage their diabetes. Their OpenAPS (Open Artificial Pancreas System) software that was created entirely by the patient community with no contribution from medical professionals automatically provides patients with the right doses of insulin based on their blood glucose level [28].

Due to the influx of advanced technologies such as wearable health sensors, portable diagnostic devices, and AI and ML applications in health care, it has become inevitable to design regulations and guidelines for technologies that are not available in the market yet, but everything, including patent trends, indicates that they will soon be. As patients now have access to technologies, data, and algorithms, they will find a way to use the technology that is not yet regulated but can still help them manage their condition or health.

The recent rise of the conversational agent and large language model ChatGPT and AI-based image generators such as MidJourney and DALL-E all point toward this direction. As a response to ChatGPT, Google LLC published a study about their own chatbot that was specifically designed to answer medical questions [29].

We expect that by looking at medical and health care–related AI and ML patent trends, regulators and policy makers could better determine medical specialties, technological trends, or areas such as imaging to dedicate more attention to. Thus, when a range of AI- and ML-based technologies become available in those fields, proper regulations will ensure a safe and efficient implementation into the practice of medicine and the delivery of health care.

A follow-up study that closely follows some of the patents and medical specialties that stood out in this analysis would be useful to see and determine how much time it takes for an AI- or ML-based health care patent to reach the stage of practical implementation.

### Limitations

There are obvious limitations to our approach. As there is no globally accepted patent database, we could only focus on the 5 most active patent offices with the highest number of patents worldwide. This implies that we might have overlooked patents from other patent offices worldwide. As there is no database in the literature about what keywords and expressions might be associated with certain medical specialties, the database we generated is a subjective list of keyword-specialty associations. Moreover, even if a specific medical specialty or its keyword is mentioned in a patent’s abstract, it does not necessarily mean that the patents are indeed associated with the specialty.

## Appendix

Multimedia Appendix 1 Medical specialties and related terms.

## Abbreviations

AI: artificial intelligence
CNIPA: China National Intellectual Property Administration
CPC: Cooperative Patent Classification
EPO: European Patent Office
FDA: US Food and Drug Administration
JPO: Japan Patent Office
KIPO: Korean Intellectual Property Office
ML: machine learning
