# Supplementary material for: Forecasting Artificial Intelligence Trends in Health Care: Systematic International Patent Analysis
Source: JMIR AI. 2023 May 26;2:e47283. doi: 10.2196/47283 (PMC11041483; doi:10.2196/47283)
Supplement: Multimedia Appendix 1 [file ai_v2i1e47283_app1.pdf]

Medical specialty

Anesthesiology	anesthesiologist	anesthesia	anesthetic
Cardiology	Echocardiogram	Cardiac	coronary
Dentistry	Cavity	caries	dental
Dermatology	epithelium	dermatological	skin
Emergency medicine	acute	emergency	
Gastroenterology	digestion	colonoscopy	endoscopy
Gerontology	aging	ageing	
Internal medicine	diabetes	metabolism	diabetic
Neurology	neurological		
OB/GYN	postpartum	pregnancy	menopause
Oncology	cancer	benign	malignant
Ophthalmology	Color blindness	myopia	retina, retinal
Pathology	biopsy free	biopsy	Cytogenetics
Pediatrics	pediatric	neonatal	baby
Primary care	family medicine	lifestyle	lifestyle medicine
Psychiatry	addiction	depression	anxiety
Pulmonology	Asthma	Bronchoscopy	COPD
Radiology	imaging	CT scan	ultrasound
Surgery	elective	minimally invasive surgery	robotic surgery
Urology	Cystitis	urological	Incontinence

Related terms

sedation				
Electrocardiogram	ECG	cardiovascular	intracardiac	post-cardiac
denture	oral health	Periodontal		
skin care	dermatoscope	dermatopathy		
gastroscopy	gastro	gastrointestinal	gastroscope	
diabetics				
trimester	labor			
chemotherapy	Carcinoma	tumor	remission	oncological
eye vision	Ophthalmological	vision		
Cytology	Cytopathology	Pathologist	histopathology	
newborn	lactation	breastfeed	pediatrician	
telehealth	telemedicine	remote care	Antibiotics	Antibiotic
mental health	telepsychiatry			
lung	ventilation	Pneumonia	pulmonological	Pneumothorax
X-ray	MRI			
laparoscopy	surgical			
Impotence	kidney	renal		

anticancer	cancerous	canceration	precancerous

Anti-microbi	anti-fungal	Disinfection	pathogenic
pulmonary			
